# Role of invasive carnivores (*Procyon lotor* and *Nyctereutes procyonoides*) in epidemiology of vector-borne pathogens: molecular survey from the Czech Republic

**DOI:** 10.1186/s13071-023-05834-w

**Published:** 2023-07-05

**Authors:** Ondřej Daněk, Paulina Maria Lesiczka, Iva Hammerbauerova, Karolina Volfova, Jana Juránková, Lucia Frgelecová, David Modrý, Kristyna Hrazdilova

**Affiliations:** 1 Department of Pathology and Parasitology, Faculty of Veterinary Medicine, University of Veterinary Sciences Brno, Brno, Czech Republic; 2 Biology Centre, Institute of Parasitology, Czech Academy of Sciences, České Budějovice, Czech Republic; 3 Department of Veterinary Sciences, Faculty of Agrobiology, Food and Natural Resources, Czech University of Life Sciences Prague, Prague, Czech Republic; 4 Department of Parasitology, Faculty of Science, Charles University, Prague, Czech Republic; 5 Department of Botany and Zoology, Faculty of Science, Masaryk University, Brno, Czech Republic; 6 Department of Chemistry and Biochemistry, Mendel University, Brno, Czech Republic

**Keywords:** Carnivores, Invasive species, Vector-borne pathogens, *Anaplasma phagocytophilum*, *Hepatozoon*, *Babesia*, *Bartonella*

## Abstract

**Background:**

Vector-borne pathogens (VBPs) are a major threat to humans, livestock and companion animals worldwide. The combined effect of climatic, socioeconomic and host composition changes favours the spread of the vectors, together with the expansion of invasive carnivores contributing to the spread of the pathogens. In Europe, the most widespread invasive species of carnivores are raccoons (*Procyon lotor*) and raccoon dogs (*Nyctereutes procyonoides*). This study focused on the detection of four major groups of VBPs namely *Babesia*, *Hepatozoon*, *Anaplasma phagocytophilum* and *Bartonella* in invasive and native carnivores in the Czech Republic, with the emphasis on the role of invasive carnivores in the eco-epidemiology of said VBPs.

**Methods:**

Spleen samples of 84 carnivores of eight species (*Canis aureus*, *Canis lupus*, *Lynx lynx*, *P. lotor*, *Martes foina*, *Lutra lutra*, *Mustela erminea* and *N. procyonoides*) were screened by combined nested PCR and sequencing for the above-mentioned VBPs targeting 18S rRNA and *cytB* in hemoprotozoa, *groEL* in *A. phagocytophilum*, and using multilocus genotyping in *Bartonella* spp. The species determination is supported by phylogenetic analysis inferred by the maximum likelihood method.

**Results:**

Out of 84 samples, 44% tested positive for at least one pathogen. Five different species of VBPs were detected in *P. lotor*, namely *Bartonella canis*, *Hepatozoon canis*, *Hepatozoon martis*, *A. phagocytophilum* and *Bartonella* sp. related to *Bartonella washoensis*. All *C. lupus* tested positive for *H. canis* and one for *B. canis*. Three VBPs (*Hepatozoon silvestris*, *A. phagocytophilum* and *Bartonella taylorii*) were detected in *L. lynx* for the first time. *Babesia vulpes* and yet undescribed species of *Babesia*, not previously detected in Europe, were found in *N. procyonoides*.

**Conclusions:**

Wild carnivores in the Czech Republic are hosts of several VBPs with potential veterinary and public health risks. Among the studied carnivore species, the invasive raccoon is the most competent host. Raccoons are the only species in our study where all the major groups of studied pathogens were detected. None of the detected pathogen species were previously detected in these carnivores in North America, suggesting that raccoons adapted to local VBPs rather than introduced new ones. *Babesia vulpes* and one new, probably imported species of *Babesia*, were found in raccoon dogs.

**Graphical abstract:**

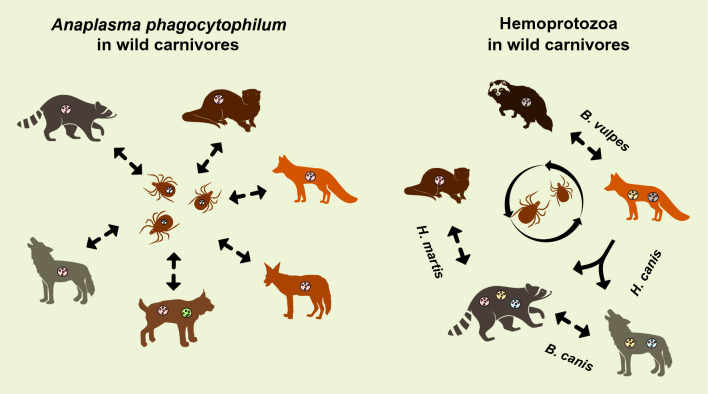

**Supplementary Information:**

The online version contains supplementary material available at 10.1186/s13071-023-05834-w.

## Background

Vector-borne diseases (VBD) represent a major public health problem as they account for more than 15% of infectious diseases worldwide [1, 2]. Wildlife and in particular wild carnivores are considered as the main source of vector-borne pathogens (VBP) for humans, livestock and companion animals [3, 4]. Domestic animals can also contribute to the bidirectional flow of VBPs between their sylvatic and domestic cycles [5]. In the last decades, some of these diseases have been encountered more frequently in Europe. The combined effect of climatic, socioeconomic and host composition changes favours the spread of the vectors [6, 7], together with the expansion of invasive carnivores contributing to the spread of the pathogens [8].

The composition of carnivores in Europe underwent changes during the nineteenth and twentieth centuries. In the last decade, all the locally extinct native species have been slowly recolonising previously occupied territories, the Czech Republic being no exception [9]. Large carnivores are mainly represented by grey wolf (*Canis lupus*) and Eurasian lynx (*Lynx lynx*), with both species being rare in the Czech Republic. Red fox (*Vulpes vulpes*) is the most prevalent mesocarnivore in the area, followed by the European badger (*Meles meles*). Small carnivores are mainly represented by species of the genus *Martes* (*Martes foina*, *M. martes*) [10].

However, by the end of the twentieth century, three new species have established their populations in Central Europe—raccoon dog (*Nyctereutes procyonoides*), common raccoon (*Procyon lotor*) and golden jackal (*Canis aureus*) [11–13]. The first two were introduced to Europe through fur farming, hunting and pet trade and are considered invasive alien species [14, 15], while the occurrence of the golden jackal is regarded as natural range expansion from the Balkans and Middle East [16]. Recent data show that these species have the potential to spread through most of the European continent [17, 18]. These carnivores with the ability to adapt to a wide variety of habitats significantly affect local ecosystems [19] and play a role in the epidemiology of zoonotic parasites and other pathogens (e.g., *Echinococcus* and *Baylisascaris*) [20, 21].

Racoons and raccoon dogs can both harbour a broad range of VBPs in both their natural and introduced habitats [8]. VBPs of zoonotic importance detected in raccoons in their natural habitat include *Anaplasma phagocytophilum* [22] and species of the genus *Bartonella*, namely *B. rochalimae* and *B. vinsonii* subsp. *berkhoffii* [23, 24]. Also, high prevalence of at least four *Babesia* spp. was detected in raccoons in the USA and Canada [25]. They also host an undescribed *Hepatozoon* sp. detected in the USA [26]. Among these pathogens, a slightly different spectrum of VBPs has been detected in the European population of raccoons. *Anaplasma phagocytophilum* was detected in one raccoon from Poland, while data on *Bartonella* in this species in Europe are missing [27]. Among the hemoprotozoa, only *H. canis* has been detected in these animals in Europe so far [28]. In racoon dogs in South Korea, a single study detected *A. phagocytophilum* [29], and zoonotic *B. rochalimae* was detected in Japan [30]. Raccoon dogs in the Far East are known hosts of *Babesia* sp. belonging to the *B. microti* group [31], but no *Hepatozoon* sp. has been found in these carnivores so far. European studies found DNA of *B. vulpes* and *A. phagocytophilum* in raccoon dogs [32, 33]. Data on *Bartonella* spp. in European raccoon dogs are missing. A number of VBPs were also detected in golden jackals, as golden jackals in Serbia were positive for *A. phagocytophilum* [34]. A study from Israel found *B. rochalimae* and *B. vinsonii* subsp. *berkhoffii* in these carnivores [35], and another study focusing on golden jackals from Romania and individuals from the Czech Republic and Austria found three species of canine hemoprotozoa—*B. canis*, *B. vulpes* and *H. canis* [36].

The most abundant and widespread native carnivore of Europe is the red fox. Foxes along with other small and mesocarnivores (e.g., European badger, martens) are actively hunted in many countries making them available for blood and tissue samples collection. Red foxes harbour important zoonotic VBPs such as *B. rochalimae* and *A. phagocytophilum* [37, 38], but they are also important hosts of *B. vulpes* and *H. canis*, both potentially pathogenic in domestic dogs [39]. Large carnivores in Europe, namely grey wolf, brown bear (*Ursus arctos*) and Eurasian or Iberian lynx, are protected throughout the European Union (EU) to some degree [40]. So far, the zoonotic bacterium *B. rochalimae* has been detected in wolves as well as canine hemoprotozoa *B. canis* and *H. canis* [41–43]. In Eurasian lynx, an undetermined *Babesia* sp. has been found in Turkey and *Cytauxzoon europaeus* in the Czech Republic and Romania [44, 45]. One study also detected *Hepatozoon felis* in ticks collected from lynx [46]. However, neither *A. phagocytophilum* nor *Bartonella* spp. have been detected from this carnivore so far.

This work was a follow-up to the study on VBPs of red foxes in the Czech Republic, which focused on the most prevalent carnivore of the country [39]. The aim of our study was to investigate VBPs of less common species of native carnivores and the invasive ones present in the Czech Republic, with an emphasis on the role of invasive carnivores in the eco-epidemiology of VBPs.

## Methods

### Study area and sampling

As part of a comprehensive survey of carnivore parasites in the Czech Republic, 84 carcasses of eight species were collected between 2014 and 2021 (Table 1), as roadkill or poached animals (in the case of protected species) or hunted animals. These were obtained in collaboration with local hunters and nature conservation agencies and examined by complete necropsy. Samples of skin, blood, heart muscle, liver, lung and trachea, spleen, kidney, muscle, and macroscopic ecto- and endoparasites were collected. Tissue samples were stored at −20 °C for further procedures. Endoparasites were morphologically identified to genus level and stored at −20 °C in 96% ethanol.

**Table 1:** Detected vector-borne pathogens and their prevalence

Host species	Total no. of animals	No. of animals positive for any pathogen	Number of positive animals/prevalence (%)							
			*Anaplasma phagocytophilum*	*Bartonella* spp.	*Babesia canis*	*Babesia vulpes*	*Babesia* sp.	*Hepatozoon canis*	*Hepatozoon silvestris*	*Hepatozoon* sp.
*Canis aureus*	3	2	1/33.3	1/33.3						
*Canis lupus*	10	10	1/10		1/10			10/100		
*Lynx lynx*	17	6	3/17.6	1/5.8					2/11.8	
*Procyon lotor*	35	12	4/11.4	6/17.4	2/5.7			1/2.9		3/8.6
*Martes foina*	2	1								1/50
*Lutra lutra*	3	1		1/33.3						
*Mustela erminea*	1	1	1/100							
*Nyctereutes procyonoides*	16	4				3/18.5	1/6.2			
Total	84	37	10	9	3	3	1	11	2	4

### DNA extraction, polymerase chain reaction (PCR) protocols and sequencing

DNA was extracted from spleen samples using a commercial kit (QIAamp DNA Blood & Tissue Kit, Qiagen, Hilden, Germany) according to the manufacturer’s instructions. The success of DNA isolation was confirmed by total DNA concentration measurement using the Qubit dsDNA [double-stranded DNA] HS Assay Kit (Thermo Fisher Scientific, USA). Samples were tested for the presence of DNA from the major groups of tick-borne pathogens.

Nested PCR assays for detection of *A. phagocytophilum*, *Babesia* spp., *Candidatus* Neoehrlichia spp. and *Hepatozoon* spp. were performed using 2× PCRBIO Taq Mix Red (PCR Biosystems, UK). First-round reactions in nested protocols were prepared in a total volume of 15 µl using 2 µl of template DNA and 0.5 μM of each primer. In the second round, 1 µl of the reaction from the first round was used as a template with 0.4 μM of each primer in a total volume of 25 µl. To determine the *groEL* ecotype of *A. phagocytophilum*, 1297-base-pair (bp) fragments of the *groEL* operon or (in the case of a missing amplicon) 407 bp of the *groEL* gene were amplified by nested PCR as previously described by Hrazdilová et al. [47].

Detection of *Bartonella* spp. was performed by amplification of multiple loci chosen based on the recommendations in La Scola et al. [48] and previous results from a *Bartonella* screening study from the Czech Republic [49] with nested PCR of citrate synthase gene (*gltA*), β subunit of bacterial RNA polymerase gene (*rpoB*), 16S-23S intergenic spacer (ITS) and repeated amplification of the *ftsZ* gene. All reactions were performed using the PPP Master Mix (Top-Bio, Vestec, Czech Republic) according to the manufacturer’s instructions. The volume of the first reaction was 15 µl, with 2 µl of template DNA, and for the second reaction 20 µl, with 1 µl of PCR product. All reactions contained 10 pmol of each primer. In all PCR assays, DNA-free water was used as negative control. Details of all PCR reactions can be found in Additional file 1: Table S1.

Amplicons were separated by electrophoresis in a 1.5% agarose gel stained with Midori Green Advance (Nippon Genetics Europe, Germany) or SYBR^®^ Gold Nucleic Acid Gel Stain (Thermo Fisher Scientific, USA) and visualised under UV light. All PCR products of the expected size were excised from the gels, purified using Gel/PCR DNA Fragments Kit (Geneaid, Taiwan) and sequenced in both directions using the amplification primers. Sequence analysis was performed by SeqMe (Czech Republic) or by Macrogen Capillary Electrophoresis Sequencing services (Macrogen Europe, the Netherlands). The sequences obtained were processed using Geneious 11.1.4 software [50] and compared with those available in the GenBank^®^ dataset by the Basic Local Alignment Search Tool (BLAST).

### Phylogenetic analysis

#### *Babesia* spp.

For 18S ribosomal RNA (rRNA) gene sequences of *Babesia* spp., the tree covering the entire order Piroplasmida was built to confirm and specify the identity and phylogenetic position of the sequences from this study. Based on this phylogeny, the detailed analysis of dog-infecting *Babesia* spp. and the closely related sequences forming a single, highly supported clade was performed. For detailed analysis, well-described species are represented by randomly chosen sequences (originating from different studies) and accompanied by all the *Babesia* sp. sequences from the same clade. Three sequences of *Babesia caballi* (the most closely related species) were used as an outgroup. The cytochrome b phylogeny was performed using all the available sequences longer than 400 bp retrieved by tBLASTx restricted to order Piroplasmida. The identical sequences were limited to five per species. Three sequences of *Plasmodium* spp. were used as an outgroup.

#### *Hepatozoon* spp.

For the phylogeny of *Hepatozoon* spp., all the available sequences of the 18S rRNA gene of the suborder Adeleorina longer than 1000 bp and unique sequences acquired in this study were used. Two sequences of *Hammondia hammondi* and one sequence of *Toxoplasma gondii* were used as an outgroup.

#### *Anaplasma phagocytophilum*

The phylogeny of *A. phagocytophilum* was constructed using unique *groEL* haplotypes detected in this study along with sequences from GenBank representing four ecotypes described by Jahfari et al. [51]. A sequence of *Anaplasma platys* was used as an outgroup. Due to unequal sequence lengths, the alignment was calculated in two steps using the MAFFT (multiple alignment using fast Fourier transform) algorithm ‘Auto’ strategy for sequences > 1000 nucleotides (nt) and the –add function for implementing sequences < 1000 nt in the alignment.

#### *Bartonella* spp.

To confirm and specify the identity and phylogenetic position of obtained *Bartonella* sequences of *gltA*, *rpoB* and ITS, the phylogeny based on all available, non-identical sequences of the respective genes in GenBank was constructed, using sequences of *Brucella abortus* as an outgroup. These served as the baseline for the detailed phylogeny of clades containing sequences obtained in this study.

The details of all the phylogenetic analyses (number of used sequences, algorithm, length of the final alignments and chosen evolution models) are listed in Additional file 1: Table S2. All the phylogenies were inferred by IQ-TREE 2.1.3, and the best-fit evolution model was selected based on the Bayesian information criterion (BIC) computed by implemented ModelFinder [52]. Branch supports were assessed by the ultrafast bootstrap (UFBoot) approximation [53] and by the Shimodaira–Hasegawa-like approximate likelihood ratio test (SH-aLRT) [54]. Trees were visualised and edited in FigTree v1.4.1 and Inkscape 0.91.

## Results

The results were based on 84 carcasses of eight carnivore species belonging to four families (Mustelidae, Canidae, Procyonidae and Felidae). Thirty-seven (44%) animals tested positive for the presence of at least one pathogen by the PCR and confirmed by sequencing (Table 1). *Hepatozoon* spp. was the most frequently detected pathogen (17/20.2%), present in *C. lupus*, *P. lotor*, *L. lynx* and *M. foina*, followed by *A. phagocytophilum* (10/10.8%) detected in *C. aureus*, *C. lupus*, *L. lynx*, *P. lotor* and *M. erminea*, and *Bartonella* spp. (9/10.7%) found in *P. lotor*, *L. lynx*, *L. lutra* and *C. aureus*. In six animals, co-infections with two different VBPs were detected. Four *Bartonella* sp.-positive *P. lotor* were also positive for *A. phagocytophilum* (*n* = 2) and *H. martis* (*n* = 2). Aside from *H. canis*, *C. lupus* also harboured *B. canis* (*n* = 1) and *A. phagocytophilum* (*n* = 1). All samples tested negative for *Ca*. Neoehrlichia spp.

### *Babesia* spp.

Overall, the PCRs targeting the 18S rRNA of piroplasmids resulted in seven positive samples for *Babesia* spp. Based on the BLAST analysis three species of genus *Babesia* were detected in this study, *B. canis* (*C. lupus* and *P. lotor*), *B. vulpes* and undescribed *Babesia* sp. (both found in *N. procyonoides*). For samples positive for *B. vulpes*, PCR targeting the *cytB* was performed, resulting in three sequences identified by BLAST analysis as *Babesia* cf. *microti* with > 99% identity. All unique sequences were submitted to the GenBank database (Additional file 1: Table S3).

**Fig. 1:**
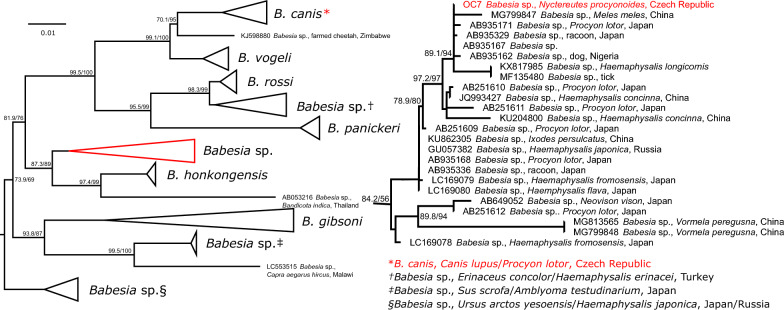
Schematic representation of the maximum likelihood phylogenetic tree based on the 18S rRNA gene sequences of *Babesia* s.s. of a dog-infecting subclade. The final length of the alignment was 1750 bp and the tree was constructed using the evolution model TPM3+F+R2. Three sequences of *B. caballi*, the closest species, used as an outgroup are not displayed. The clade in a sister position to *B. honkongensis* is shown in detail. Sequences from this study are marked in red. The scale bars indicate the number of nucleotide substitutions per site. The bootstrap values (SH-aLRT/UFB) above the 80/95 threshold are displayed. Sequences are labelled by accession number, host and country of origin (if available)

The phylogenetic analyses of the 18S rRNA gene of the entire order Piroplasmida resulted in a tree with an overall topology published previously [55] and confirmed the identity of *B. canis* and *B. vulpes* (data not shown). The *Babesia* sp. from *N. procyonoides* was placed within the clade of *Babesia* sensu stricto (clade X sensu Jalovecka et al. 2019) forming a monophyletic group with dog-infecting babesias. In in-depth analysis of this clade, a single sequence from *N. procyonoides* fell to the clade in a sister position to *Babesia honkongensis*, consisting of undescribed *Babesia* sp. originating mainly from smaller carnivores and hard ticks from Asia (Fig. 1. and Additional file 2: Fig.S1).

The identity of *B. vulpes* sequences detected in *N. procyonoides* was additionally supported by the phylogeny of *cytB* (Additional file 2: Fig. S2), placing them into the clade of *Babesia* cf. *microti*/*B. vulpes*.

### *Hepatozoon* spp.

Seventeen sequences of *Hepatozoon* spp. were obtained in this study. Based on BLAST analysis these were identified as *H. canis* (*C. lupus* and *P. lotor*, three unique haplotypes), *H. silvestris* (*L. lynx*) and *H. martis* (*P. lotor* and *M. foina*, identical sequence). One sequence from *P. lotor* was closely related to *H. martis* (98.78% sequence identity to sequence OM256569 from *M. foina* from Hungary). All unique sequences were submitted to the GenBank database (Additional file 1: Table S3).

**Fig. 2:**
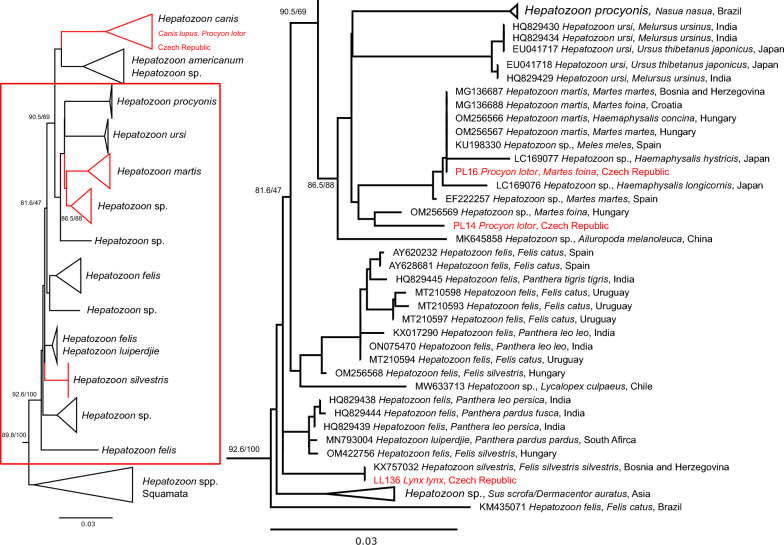
Schematic representation of the maximum likelihood phylogenetic tree based on the 18S rRNA gene sequences of the suborder Adeleorina, focused on a well-defined clade of mammal-infecting *Hepatozoon* spp. The final length of the alignment was 2139 bp and the tree was constructed using the evolution model GTR+F+R4. Two sequences of *Hammondia hammondi* and one sequence of *Toxoplasma gondii* used as an outgroup are not displayed. The cluster of clades containing sequences of *H. martis*, *H. silvestris*, *H. felis* and *H. ursi* is shown in detail. Sequences from this study are marked in red. The scale bars indicate the number of nucleotide substitutions per site. The bootstrap values (SH-aLRT/UFB) above the 80/95 threshold are displayed. Sequences are labelled by accession number, host and country of origin (if available)

The phylogeny of suborder Adeleorina (Additional file 2: Fig. S3) showed that all sequences acquired in this study belong to a well-defined clade of *Hepatozoon* spp. infecting mammalian hosts, mainly of order Carnivora. Eleven samples were confirmed as *H. canis*, two as *H. silvestris*. Sequences originating from two *P. lotor* and one *M. foina* fall within the clade of *H. martis* or its sister clade of *Hepatozoon* sp. both composed from sequences obtained mainly from mustelids (Fig. 2).

### *Anaplasma phagocytophilum*

Nested PCR resulted in 10 sequences (333–960 bp). Six unique haplotypes (V1–V6), characterised by 95.08–99.87% sequence similarity, were detected. The most common variant (V1) was detected in three animals (*P. lotor*, *C. lupus*, *M. erminea*), variants V2 and V3 were found in two individuals each (V2: *C. aureus*, *P. lotor* and V3: *L. lynx* and *P. lotor*). The remaining three haplotypes were represented by a single individual (V4: *P. lotor*; V5: *L. lynx*; V6: *L. lynx*). Unique sequences were submitted into the GenBank database (Additional file 1: Table S3).

**Fig. 3:**
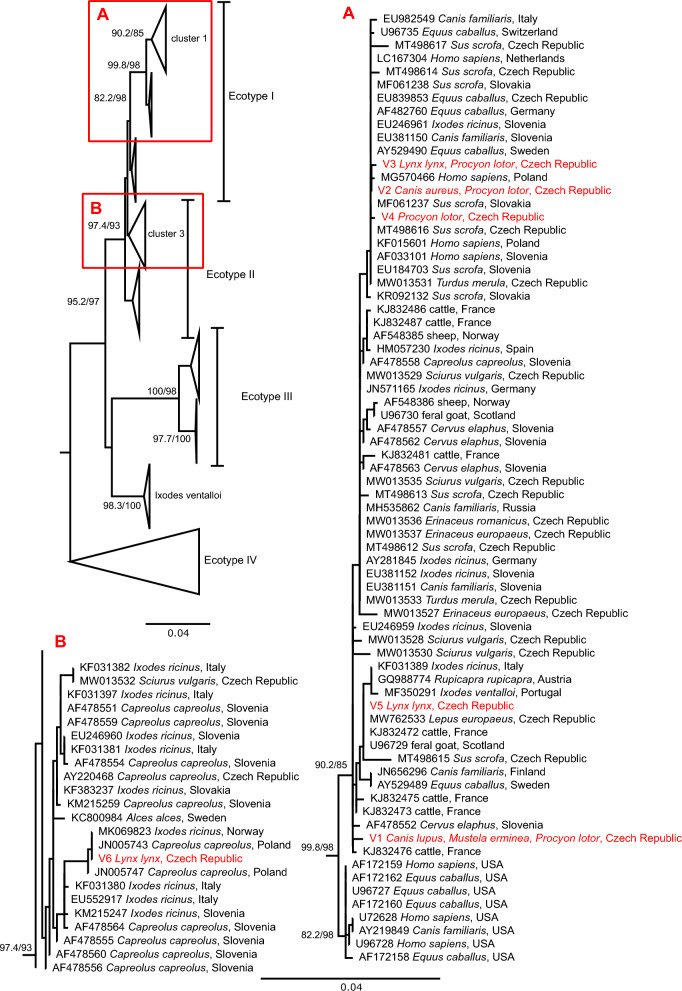
Schematic representation of the maximum likelihood phylogenetic tree based on the *groEL* gene sequences of *Anaplasma phagocytophilum*. The final length of the alignment was 1256 bp and the tree was constructed using the evolution model TN+F+I+R2. Three sequences of *A. platys* used as an outgroup are not displayed. Detailed clades belonging to ecotype I **(A)** and ecotype II **(B)** as described by Jahafari et al. (2014) are shown. Sequences from this study are marked in red. The scale bars indicate the number of nucleotide substitutions per site. The bootstrap values (SH-aLRT/UFB) above the 80/95 threshold are displayed. Sequences are labelled by accession number, host and country of origin (if available)

In the phylogenetic analysis, we followed the classification of *A. phagocytophilum* based on the partial *groEL* gene sequences, introduced by Jahfari et al. [51] and extended by Jaarsma et al. [56]. Variants V1–V5 clustered in the clade representing European ecotype I. Variants V2–V4 were grouped in the subclade with the European human cases and strains from horses, wild boars, ticks and carnivores. Variants V1 and V5 grouped among sequences coming mostly from different ungulates, carnivores and *Ixodes* ticks. The unique genotype V6 belonged to cluster-3 within ecotype II formed by sequences from *Capreolus capreolus* and *Ixodes ricinus* (Fig. 3).

### *Bartonella* spp.

Four species of carnivores tested positive for *Bartonella* spp.—one Eurasian lynx (5.9%), one European otter (33.3%), one golden jackal (33.3%) and six raccoons (17.1%). PCRs amplified product for multiple loci only in the case of otter (*gltA*, *rpoB*, ITS) while in other species PCR for only a single gene was positive—*gltA* for Eurasian lynx and raccoon and ITS for golden jackal. In the case of raccoons, all six sequences of *gltA* were identical. According to BLAST analysis, partial *gltA* sequence from Eurasian lynx was identified as *Bartonella taylorii* with 99.39 to 100% sequence identity to three nearest hits (CP083693, CP083444 and MZ680430) isolated from rodents in western Europe, while no other sequences could be identified to species level, resulting in the need for phylogenetic analyses. All unique sequences were uploaded into the GenBank database (Additional file 1: Table S3).

The phylogenetic tree of all the unique available *gltA* sequences placed sequences generated in this study into two clades. The sequence from the Eurasian lynx was confirmed as clearly belonging to one of the *B. taylorii* clades (data not shown). The sequences from raccoons and the European otter clustered together with sequences described as *B. washoensis* and *B. jaculi*. A detailed phylogeny of this clade (Additional file 2: Fig. S4) showed a close relation of sequence from *L. lutra* to a subclade of *B. washoensis* originating mainly from ground squirrels of the tribe Marmotini (and their ectoparasites), while the sequence from *P. lotor* clustered to a subclade of *B. washoensis* isolated mainly from squirrels (subfamily Sciurinae) and a subclade of sequences from different bats and their ectoparasites. Similarly, the phylogenetic tree of all the unique available *rpoB* sequences placed the sequence obtained from *L. lutra* into a clade containing *Bartonella washoensis* and *B. jaculi* (data not shown). The detailed phylogeny of this clade (Additional file 2: Fig. S5) placed the sequence from *L. lutra* into a subclade of *Bartonella* sequences from different mustelids in a sister position to a subclade of *B. washoensis* originating from ground squirrels (tribe Marmotini) and their ectoparasites. The phylogenetic analysis of all the unique available sequences of ITS placed the sequences from European otter and golden jackal into clades of sequences containing *B. washoensis* and *B. grahamii*, respectively (data not shown). The in-depth analysis of these clades placed the sequence from *L. lutra* into a subclade of sequences from different mustelids between two subclades of *B. washoensis* similarly to both previous genes (Additional file 2: Fig. S6). The sequence from *C. aureus* fell into a subclade within the *B. grahamii* clade (Additional file 2: Fig. S7). This entire clade is composed mainly of sequences obtained from rodents of families Cricetidae and Muridae (or their ectoparasites).

## Discussion

Wild carnivores are hosts of a wide variety of infectious agents, from which VBPs pose a significant health risk for humans, companion animals and livestock [57]. Due to socioeconomic and landscape changes in Europe carnivores expand through the continent, either due to recolonisation (wolves), natural range extension (golden jackals) or as invasive species (raccoons and raccoon dogs) [58–60]. The range expansion of such carnivores can contribute to the spread of VBPs [61].

### *Babesia* spp.

Carnivore-infecting piroplasmid of genus *Babesia* from both clades *Babesia* s.s. and *Babesia microti*-like group [55] were detected in this study. From *Babesia* s.s., two species were detected—*B. canis* and *Babesia* sp. forming a sister clade to *B. honkongensis*. *Babesia canis* was detected in a wolf and raccoons. Data on *B. canis* in grey wolves are scarce, with previous detection in wild [43] and captive animals, where fatal cases of babesiosis are described [62]. Recently, the presence of *B. canis* was confirmed in its vector, *Dermacentor reticulatus* [63], as well as by the first autochthonous clinical case in a dog in the Czech Republic [64]. However, this is the first detection of *B. canis* in wild carnivores in the Czech Republic, suggesting wolves may contribute to the sylvatic cycle of the parasite. In the invasive European population of *P. lotor*, *B. canis* is the first detected *Babesia*. In the USA and Japan, several unnamed *Babesia* species from both clades have been detected in raccoons so far [25, 65]. Based on phylogeny, at least two of them are shared between native and invasive populations of raccoons, showing the spread of VBPs with the carnivore [25].

A *Babesia* sp. from a clade belonging to *Babesia* s.s. was detected in a raccoon dog. This clade is composed of sequences originating from the Far East, except for two sequences from dogs from Nigeria [66]. Published sequences were isolated mostly from invasive raccoons [65] or local ticks [67, 68], with European badger [69] and marbled polecat (*Vormela peregusna*) [70] being the only native Asian carnivores in which this *Babesia* species has been reported so far. We hypothesise that this species of *Babesia* is adapted to native carnivores of the Far East, and raccoon dogs could be regular hosts. Furthermore, this species was isolated from *Haemaphysalis concinna* and *Ixodes persulcatus*, hard ticks present both in the Far East and in the European part of Russia [71, 72], meaning this species could have been imported with raccoon dog and the vector was already present, similarly to avian malaria in Hawaii [73] or *Babesia* from *B. microti*-like group possibly imported with raccoons to Japan [25]. Further studies targeted at this particular species are necessary to confirm its origin and a natural reservoir.

The only detected species of the *B. microti* group in this study was *B. vulpes* found in raccoon dogs. Based on phylogenetic analysis of previously published sequences, *B. vulpes* was also detected in native (South Korea) and invasive (Austria) populations of raccoon dogs [31, 32]. The presence of *B. vulpes* in raccoon dogs is no surprise as it is the second most prevalent VBP detected in foxes in the Czech Republic [39]. We also confirmed the identity of the isolate using the mitochondrial marker *cytB*, as it proved powerful for the clear delineation of closely related piroplasmid species [45, 74]. The use of the 18S rRNA gene sequence alone can be problematic in future, especially with a growing number of *B. microti*-like isolates from different hosts and geographical localities [75].

### *Hepatozoon* spp.

The finding of *H. canis* in all wolves in this study is consistent with the situation throughout Europe [42, 76, 77]. This parasite circulates in the area in a fox population, where *H. canis* was the most prevalent pathogen detected in the Czech Republic [39]. A single raccoon positive for *H. canis* corresponds with results from Spain [78], *H. canis* being the only detected species of *Hepatozoon* in raccoons in Europe so far. The role of both species of carnivores in the epidemiology of *H. canis* in the Czech Republic is probably minor, compared to the total number of red foxes.

Several species of *Hepatozoon* have been detected in felids so far, with the most important ones in Europe being *H. felis* and *H. silvestris* [79–81]. The two Eurasian lynx in this study positive for *H. silvestris* are the first case of *Hepatozoon* detected in these carnivores. As this species is usually detected in wild cats (*Felis silvestris*), it is likely that Eurasian lynx and wild cats share the vector of this pathogen as they share their habitats in the Czech Republic [9, 82]. Both positive Eurasian lynxes tested negative for *C. europaeus* in a previous study [45].

Despite a low number of tested mustelids, one *M. foina* was positive for *H. martis*, a species infecting European mustelids [81]. *Hepatozoon martis* was also found in two raccoons, being the first record of *H. martis* in raccoons. The sequence obtained from raccoon ID PL14 was closely related to this species, but more data is necessary to determine the exact species of *Hepatozoon* in PL14. Interestingly, *H. procyonis* was described from raccoons in the USA [83] but it has not been molecularly characterised from this species. However, *H. procyonis* was later redescribed in a different species, the South American coati (*Nasua nasua*) [84]. Published data suggest that species of *Hepatozoon* found in carnivores might not be strictly host specific as both *H. canis* and *H. martis*, pathogens of canids and mustelids respectively have been previously detected in cats [79, 81]. This suggests that raccoons could be involved in the circulation of *H. canis* and *H. martis* in Europe.

### *Anaplasma phagocytophilum*

The most prevalent bacterial VBP in our study is *A. phagocytophilum*, being the pathogen with the widest host spectrum as well. Based on phylogenetic studies of *A. phagocytophilum*, several genetic variants are recognised, such as ecotypes based on the *groEL* gene [51, 56]. Five of six detected haplotypes of *A. phagocytophilum* belonged to ecotype I. In Europe, ecotype I is the most common variant of *A. phagocytophilum* and a host generalist, being detected in at least five different mammal orders so far. Consequently, *A. phagocytophilum* infections in livestock, companion animals and humans in Europe are typically caused by this variant [85, 86]. Haplotypes of *A. phagocytophilum* detected in carnivores usually fall into cluster 1 of ecotype I [27, 87]. This study also shows that raccoons are adapted to carry European variants of *A. phagocytophilum*. Due to their synanthropic nature and frequent use of tree holes and burrows of other animal species raccoons can be infested with both questing and endophilic ticks, potentially bridging the enzootic cycles of *A. phagocytophilum* [88, 89].

The only haplotype falling into ecotype II of *A. phagocytophilum* was detected in the Eurasian lynx. Ecotype II is predominantly found in roe deer or moose (*Alces alces*) and is transmitted by *I. ricinus* in populations of these ungulates [90]. As roe deer is the main prey item of Eurasian lynx in the Czech Republic, it is most likely an accidental host of this ecotype, similar to the other non-ungulate hosts (e.g., red squirrel), but more data are needed to elucidate the enzootic cycles and host specificity of each ecotype [91, 92].

### *Candidatus* Neoehrlichia spp.

A relatively newly studied bacterial VBP of wild carnivores are species of *Ca*. Neoehrlichia spp. Raccoon dogs and foxes have been known to harbour *Ca.* Neoehrlichia sp., with its presence confirmed in the Czech Republic and neighbouring Poland [27, 39]. None of the carnivores in our study tested positive for this pathogen, which could be caused by a relatively low number of tested raccoon dogs. The overall abundance of the pathogen reported so far in the Czech Republic is also low in the main reservoir, the red fox [39].

### *Bartonella* spp.

One of the most studied groups of VBPs in the last decade are species of the genus *Bartonella*. Some species are adapted to humans or have zoonotic potential [93]. Genotyping of *Bartonella* spp. is challenging and usually based on multilocus genotyping [48, 94]. As we were not able to amplify more than one gene for most of the positive animals, our results are debatable. The most closely related species to the detected haplotypes of *Bartonella* spp. from this study were *B. washoensis*, *B. taylorii* and *B. grahamii*. All three of these species are primarily associated with rodent hosts; however, *B. washoensis* and *B. grahamii* have zoonotic potential and were also previously detected in synanthropic small animals in the Czech Republic [49, 95, 96].

The *Bartonella sp.* detected in a European otter falls into a clade of sequences composed of *B. washoensis* and *B. jaculi* and their phylogenetic positions correlate in all three sequenced genes. *Bartonella washoensis* is detected almost exclusively in rodents of the family Sciuridae, and *B. jaculi* has been detected in rodents of the genus *Jaculus* [97, 98]. Sequences from a European otter are closely related to a clade of *B. washoensis* isolated mainly from ground squirrels from North America and China. However, they are always separated in all genes and even form a subclade with sequences from a Japanese marten (*Marten melampus*, *rpoB* and ITS) and a North American river otter (*Lontra canadensis*, ITS). While these could be accidental infections, closely related sequences were found in three mustelid hosts on three different continents. This could potentially mean a variant or subspecies found in mustelids, closely related to *B. washoensis*. Only other studied *Bartonella* spp. in otters were detected in sea otters (*Enhydra lutris*) in which haplotypes related to *Bartonella henselae*, *B. tamiae* and *B. washoensis* were detected based on ITS [99]. However, these sequences are not available and could not be included in our phylogeny. The haplotype of *Bartonella* sp. obtained from raccoons is related to a clade of *B. washoensis* isolated mainly from squirrels of genus *Sciurus* from Eurasia (based on *gltA* only). While the haplotype is well separated from other published sequences and may represent a new subspecies, no conclusions should be made based the on phylogeny of a single gene. Raccoons are known hosts of *Bartonella* spp. in North America, where *B. rochalimae* and *B. vinsonii* were detected [23, 24]. Another study from the USA found the DNA of *B. henselae* and *B. koehlerae* in raccoons; however, the results are based on ITS only and no sequences are available [100]. Further research is necessary to determine the role of raccoons in the epidemiology of the detected, potentially zoonotic *Bartonella* species.

*Bartonella taylorii* is almost exclusively found in rodents, with one documented case of a raccoon from Canada with clinical disease attributed to this species [101, 102]. For this reason, we believe that our finding of a *gltA* sequence closely related to *B. taylorii* in Eurasian lynx is also an accidental infection. Although this is the first detected *Bartonella* in Eurasian lynx, related wild felids are known hosts of *B. koehlerae* and *B. henselae* [103].

The sequence of the ITS gene obtained from the golden jackal falls into a clade of sequences of *B. grahamii*. This species is predominantly found in small rodents, and we assume the carnivore was infected accidentally [101]. Golden jackals are known to harbour *B. rochalimae* [35], species found in other wild canids.

As shown in our study a detailed phylogeny should be used for species determination rather than BLAST analysis and percentage of sequence similarity. Also, the criteria for species determination proposed by La Scola et al. [48] must be considered with regard to said phylogeny and the fact that the number of recognised *Bartonella* species nearly tripled since then and more closely related species are recognised nowadays [93, 104]. Lastly, the presence of DNA of a pathogen in the spleen of an animal does not necessarily mean infection with said pathogen and the results should always be viewed in light of this considering the bigger picture [105].

## Conclusions

Among all the studied carnivore species, only raccoons harboured all the major groups of studied pathogens. None of the detected pathogen species (ecotypes of *A. phagocytophilum*) were previously found in these carnivores in North America, suggesting that raccoons adapted to local VBPs rather than introduced new ones. While species of genera *Babesia* and *Hepatozoon* may not be strictly host-specific, raccoons represent an entirely new taxonomic family of hosts [81, 106]. Also, the raccoon’s relatively high prevalence of *Bartonella* sp. closely related to zoonotic *B. washoensis* and zoonotic ecotype I of *A. phagocytophilum* requires further investigation in light of their synanthropic ecology. Raccoon dogs may play a role in the spread of *Babesia* sp. not native to Europe. However, with *B. vulpes* as the only other detected pathogen, their role in the spread of European VBPs is minor. This is the second detection of *B. canis* in free-ranging grey wolves and the first detection of *H. silvestris* in Eurasian lynx, with an unknown effect on host fitness in both protected species. Further studies are necessary to understand the role of carnivores, namely the invasive raccoons in the eco-epidemiology of VBDs.

## Supplementary Information

**Additional file 1: Table S1** Protocols of all PCRs used in this study. **Table S2** The details of all the phylogenetic analyses. **Table S3 **Details of sequences uploaded into the GenBank database.

**Additional file 2: Fig. S1–S7** Supplementary phylogenetic trees.

## Data Availability

The nucleotide sequence generated in the present study has been deposited in GenBank (https://www.ncbi.nlm.nih.gov/), with detailed information present in Additional file 1: Table S3. The datasets used and/or analysed during the current study are available from the corresponding author on reasonable request.
