# Supplementary material for: Role of invasive carnivores (Procyon lotor and Nyctereutes procyonoides) in epidemiology of vector-borne pathogens: molecular survey from the Czech Republic
Source: Parasit Vectors. 2023 Jul 5;16:219. doi: 10.1186/s13071-023-05834-w (PMC10324142; doi:10.1186/s13071-023-05834-w)
Supplement: Supplementary file 2 — Additional file 2: Fig. S1–S7 Supplementary phylogenetic trees. [file 13071_2023_5834_MOESM2_ESM.zip › Additional file 2/Fig_S2_Babesia_cytB_rev.pdf]

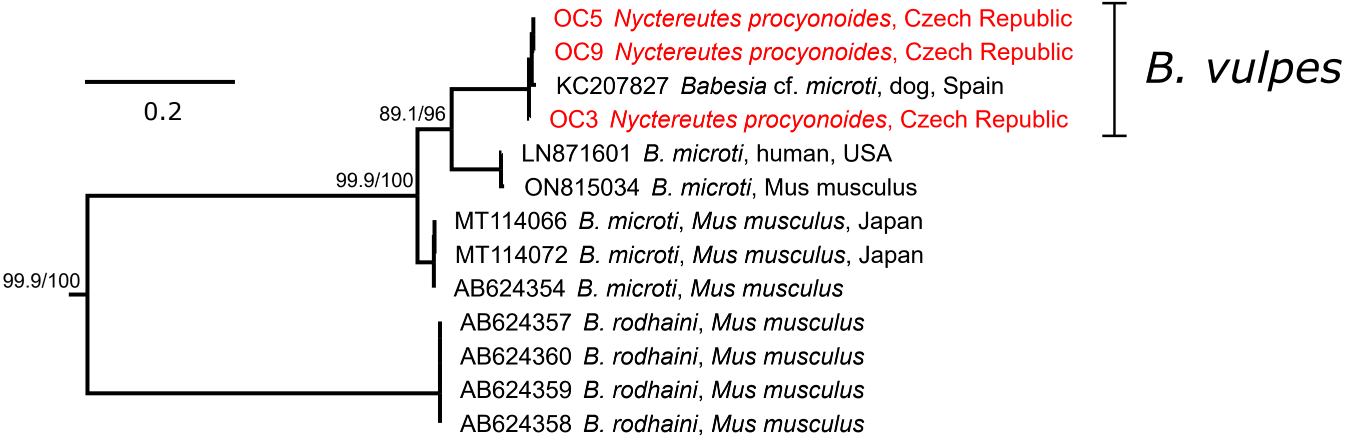

Fig S2. Schematic representation of the maximum likelihood phylogenetic tree based on the *cytB* gene sequences of order Piroplasmida; *Babesia microti*-like clade shown in detail. The final length of the alignment was 1296 bp and tree was constructed using evolution model TIM+F+I+G4. Three sequences of *Plasmodium* spp. used as an outgroup are not displayed. Sequences from this study are marked in red. The scale bars indicate the number of nucleotide substitutions per site. The bootstrap values (SH-aLRT/UFB) above the 80/95 threshold are displayed. Sequences are labelled by accession number, host, and country of origin (if available).
