# Supplementary figures and images for: Role of invasive carnivores (Procyon lotor and Nyctereutes procyonoides) in epidemiology of vector-borne pathogens: molecular survey from the Czech Republic
Source: Parasit Vectors. 2023 Jul 5;16:219. doi: 10.1186/s13071-023-05834-w (PMC10324142; doi:10.1186/s13071-023-05834-w)

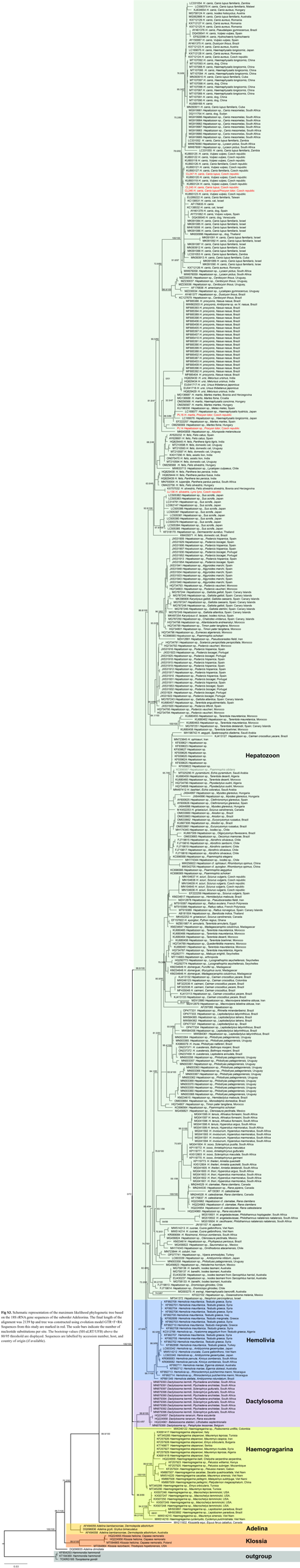

Supplement: Supplementary file 2 — Additional file 2: Fig. S1–S7 Supplementary phylogenetic trees. [file 13071_2023_5834_MOESM2_ESM.zip › Additional file 2/Fig_S3_Hepatozoon_18S_rev.pdf]

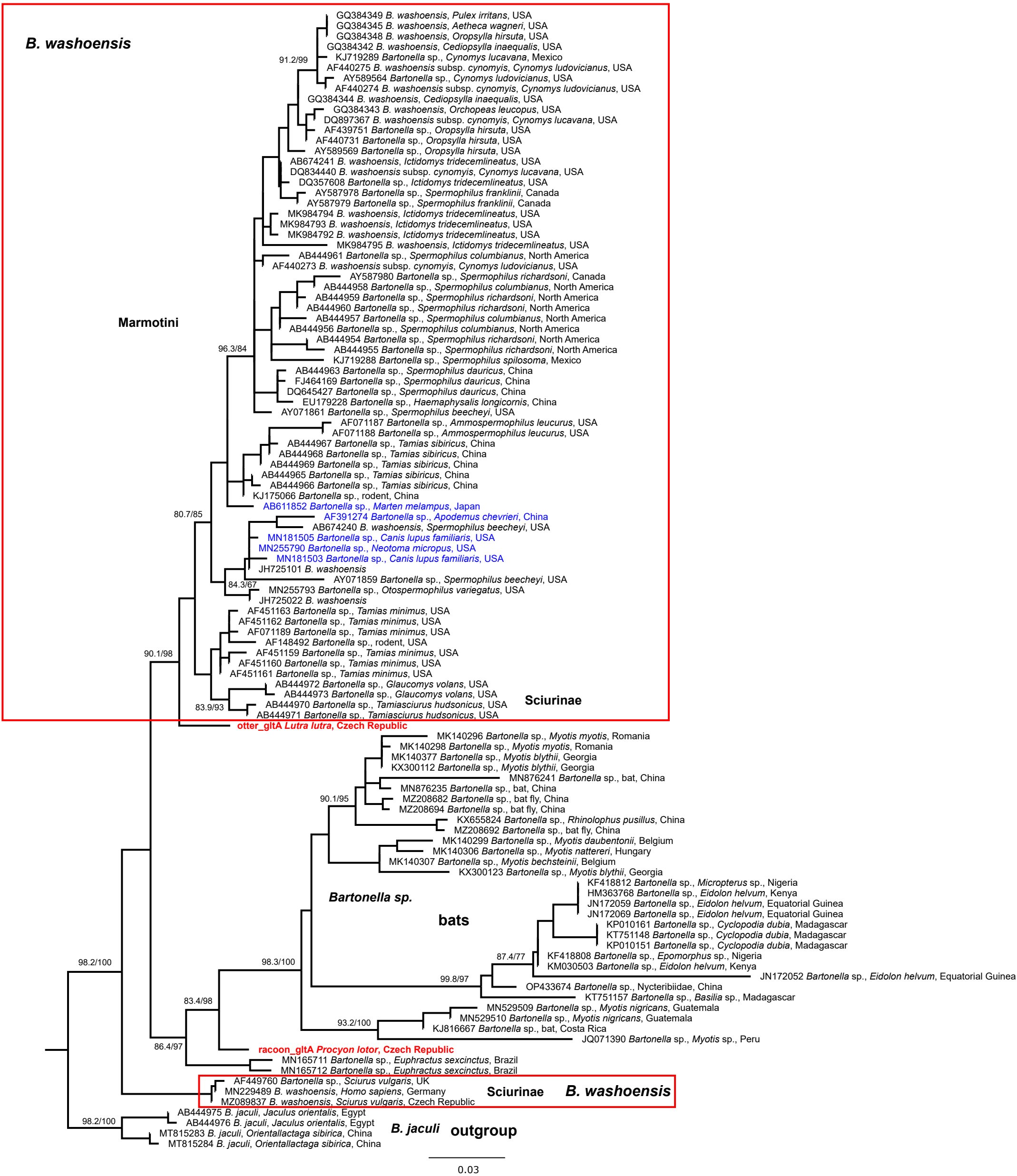

Supplement: Supplementary file 2 — Additional file 2: Fig. S1–S7 Supplementary phylogenetic trees. [file 13071_2023_5834_MOESM2_ESM.zip › Additional file 2/Fig_S4_Bartonella_gltA_rev.pdf]
